# Strangulation du colon sigmoïdien par un testicule ectopique géant

**Published:** 2012-03-28

**Authors:** Zimogo Sanogo, Adama Koita, Moussa Camara, Lamine Soumaré, Bakarou Kamaté, Dieneba Doumbia, Zanafon Ouattara, Ali Tembely, Sadio Yena, Youssouf Coulibaly, Djibril Sangaré

**Affiliations:** 1 Service de chirurgie A, hôpital du POINT G Bamako, Mali; 2 Service d’urologie, hôpital du POINT G Bamako, Mali; 3 Service d’anatomopathologie, hôpital du POINT G, Bamako, Mali; 4 Service d’anesthésie et réanimation, hôpital du POINT G Bamako, Mali

**Keywords:** Volvulus, colon sigmoïde, cryptorchidie, testicule, Mali

## Abstract

Les causes de volvulus du colon sigmoïde sont variées et parmi elles la strangulation est des plus fréquentes dans notre contexte d’exercice. Les lésions vues tard permettent très rarement un traitement sans résection. La survenue d’un volvulus du colon sigmoïde autour d’un pédicule de testicule géant ectopique est une première que nous rapportons dans cette étude de cas.

## Introduction

Les occlusions par strangulations et particulièrement par volvulus du sigmoïde sont l’une des premières causes d’urgences chirurgicales à l’hôpital du Point G, Bamako et dans la sous-région [1,2]. Le cas clinique rapporté est une particularité. Les signes classiques d’occlusion étaient associés à une tuméfaction pelvienne palpable mobile. Le diagnostic de volvulus du colon sigmoïdien autour du pédicule d’un volumineux testicule ectopique gauche a été posé en per opératoire. Une ligature-résection du pédicule testiculaire et une résection-anastomose du sigmoïde en un temps ont été pratiquées. Les suites opératoires ont été simples. Le but de cette étude était de rapporter les éléments cliniques et thérapeutiques du cas clinique.

## Patient et observation

GA, un patient de 29 ans était admis en urgence à l’hôpital du Point G Bamako, Mali. Le motif de consultation était une douleur abdominale péri ombilicale à début brutal sans irradiations et sans aucune tendance à la rémission. La douleur était accompagnée de nausées et de vomissements et d’un arrêt franc des matières et des gaz. L’examen clinique du patient permettait de découvrir à l’inspection un abdomen tendu, des mouvements de péristaltisme intermittents et une asymétrie abdominale. A la palpation l’abdomen était douloureux dans la fosse iliaque gauche, siège d’une masse lisse, arrondie dont la mobilisation facile exacerbait la douleur. La percussion révélait un tympanisme maximal dans l’hypochondre droit, mais aussi la douleur. La bourse testiculaire gauche était vide, celle de droite était occupée par un testicule de taille normale. L’ampoule rectale était vide au toucher rectal.

La radiographie de l’abdomen sans préparation permettait de retrouver des images de niveaux hydro-aériques plus hauts que larges, en cadre dans la fosse iliaque gauche et dans l’hypochondre gauche.

**Figure 1:**
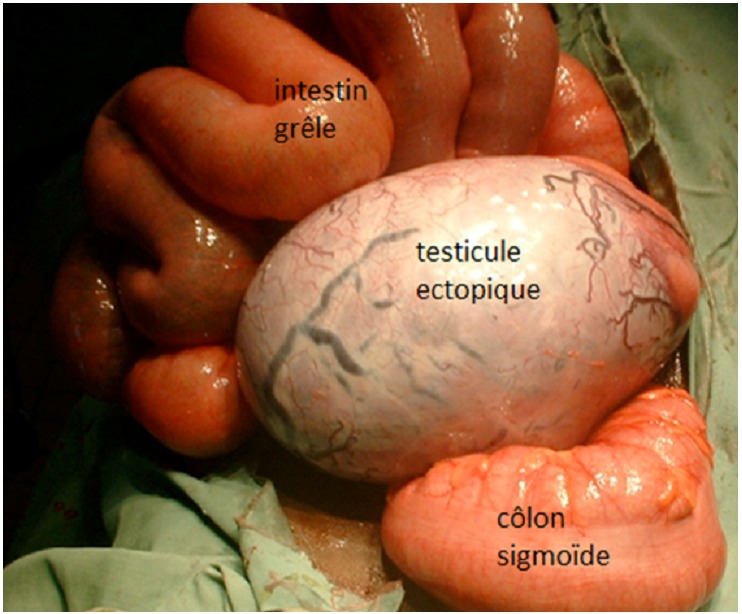
Intestin grêle, testicule ectopique et colon sigmoïdien

**Figure 2:**
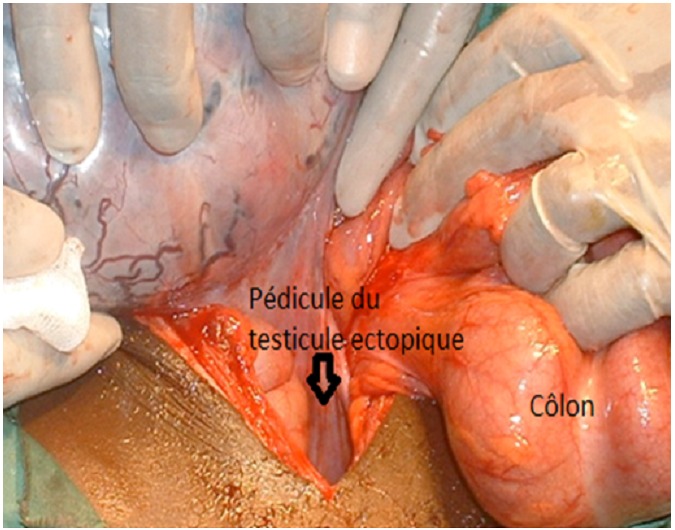
Pédicule du testicule ectopique

La laparotomie sous anesthésie générale permettait la découverte dans le pelvis à l’exploration une masse d’environ 10 cm de grand axe, de la forme d’un œuf, pédiculée. Le long pédicule de la masse cravatait au pied la boucle de colon sigmoïde en semi torsion autour de l’obstacle. Il n’existait aucune nécrose du colon étranglé. Il n’existait pas d’adénomégalie intra-abdominale. Les segments coliques d’amont et d’aval et les autres organes intra péritonéaux étaient sans anomalie macroscopique. La masse d’aspect blanc nacrée correspondait à un testicule ectopique gauche géant intra-abdominal (Figure 1). Le pédicule testiculaire était libéré par résection et ligature à la base Figure 2, et la boucle colique sigmoïdienne reséquée. La continuité colique était rétablie en un temps par anastomose termino-terminale colo-colique et la paroi abdominale fermée plan par plan. Les suites opératoires furent simples et la sortie de l’hôpital eut lieu à J10. L’examen histologique du testicule géant montrait un tissu gonadique sans atypie cellulaire.

## Discussion

**Figure 3:**
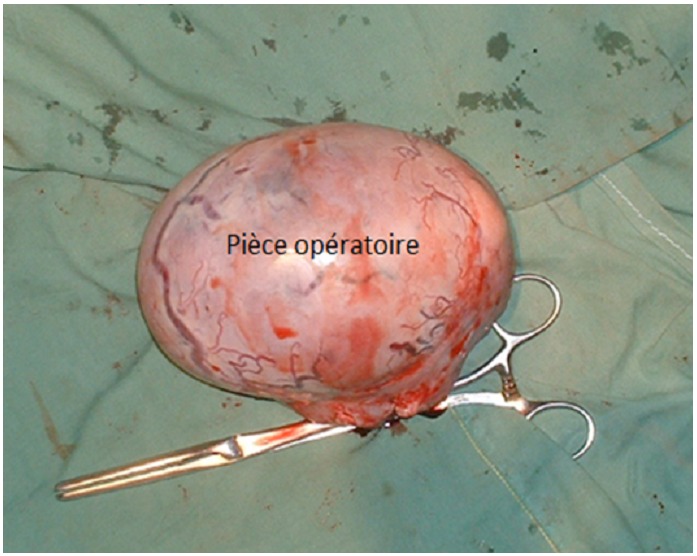
La pièce opératoire mesurant 10 cm de grand axe

Il s’agit d’un cas d’observation rare d’occlusion par strangulation. Les causes de ce type d’occlusion sont avant tout dans notre contexte d’exercice les hernies pariétales inguinales et fémorales mais aussi le volvulus du colon sigmoïde [2]. Les signes cliniques fonctionnels ont été les vomissements tardifs classiques de l’occlusion basse, la douleur abdominale de survenue brutale chez un sujet jusque là en bonne santé, l’arrêt des matières et des gaz précoce. L’examen physique trouvait en plus des signes classiques (météorisme, tympanisme asymétrie abdominale) une tuméfaction palpable de la fosse iliaque gauche. Le diagnostic préopératoire d’occlusion sur tumeur de la jonction recto-sigmoïdienne a été évoqué. La taille d’environ 10 cm de la tuméfaction (Figure 3), son aspect lisse et arrondi, sa mobilité surtout l’apparition brutale de la symptomatologie n’étaient pas en faveur du diagnostic de tumeur maligne. En outre le bon état général du malade et son jeune âge plaidaient contre ce diagnostic. Autre diagnostic différentiel évoqué, l’abcès a été exclu du fait de l’absence de fièvre. En per opératoire la présence de la tuméfaction de couleur blanc-nacrée arrondie et pédiculée a conduit à revoir la vacuité effective de la bourse testiculaire gauche notée déjà dans l’anamnèse.

**Figure 4:**
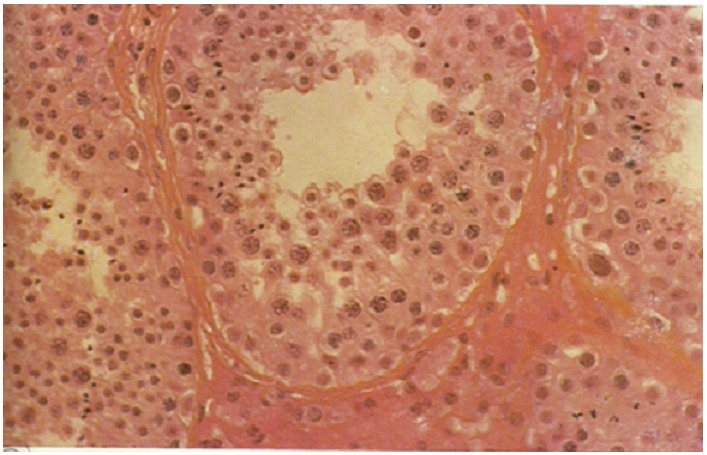
Coupe microscopique du testicule ectopique, absence de cellules pathologiques à l’examen

Le volumineux testicule en position abdominale a été réséqué après ligature première de la veine, de l’artère et du canal déférent. La dégénérescence maligne du testicule en position ectopique est corrélée avec un risque de cancérisation élevé [3]. Ce risque serait d’autant plus grand que l’âge du sujet est avancé. La résection anastomose du colon sigmoïde en un temps a été dictée par le jeune âge, l’aspect propre du colon et l’absence de nécrose ou de perforation, comme d écrite par Bufin [4]. L’absence d’adénomégalie pelvienne et inguinale rassurait quant à une éventuelle cancérisation. Cependant le protocole habituel de recherche à froid de cellules malignes sur les pièces opératoires a été respecté. Il n’existait pas de dégénérescence maligne du testicule ectopique géant et du colon sigmoïde (Figure 4).

## Conclusion

L’occlusion par strangulation est fréquente, mais la strangulation par un pédicule de testicule ectopique demeure rare. Le diagnostic d’occlusion posé tôt a permis d’éviter les complications liées à une strangulation de longue durée pouvant aboutir à la nécrose et à la perforation colique.
